# Biocide Tolerance, Biofilm Formation, and Efflux Pump Activity in Clinical Isolates of *Trichosporon asahii*

**DOI:** 10.3390/idr17040097

**Published:** 2025-08-06

**Authors:** Yasmim Passos Lima, Jamile de Paiva Macedo, Alessandra Barbosa Ferreira Machado, Cláudio Galuppo Diniz, Vania Lucia da Silva, Vanessa Cordeiro Dias

**Affiliations:** Department of Parasitology, Microbiology, and Immunology, Federal University of Juiz de Fora, Rua José Lourenço Kelmer, s/n, São Pedro, Juiz de Fora 36036-900, MG, Brazil; yasmimpassos14@gmail.com (Y.P.L.); jamile_paiva_macedo@hotmail.com (J.d.P.M.); alessandra.machado@ufjf.br (A.B.F.M.); claudio.diniz@ufjf.br (C.G.D.); vania.silva@ufjf.br (V.L.d.S.)

**Keywords:** *Trichosporon asahii*, infection, biofilm, efflux pump, biocides tolerance, sodium hypochlorite

## Abstract

Background: *Trichosporon* spp. are opportunistic fungi, capable of causing infection, especially in critically ill individuals who often use broad-spectrum antibiotics, invasive devices, and have comorbidities. Objectives The aim of this study was to analyze individuals’ clinical characteristics, evaluate tolerance to biocides, as well as biofilm formation and efflux pump activity in isolates of *Trichosporon asahii*. Methods: Clinical isolates of *T. asahii* collected between 2020 and 2023 from both hospitalized and non-hospitalized individuals, of both sexes, regardless of age, were tested for tolerance to sodium hypochlorite, hydrogen peroxide, benzalkonium chloride, and ethyl alcohol. Efflux pump activity was also assessed using ethidium bromide, and biofilm formation was measured with the Safranin test. Clinical parameters such as outcomes, source, and length of hospitalization were analyzed through electronic medical records. Results: A total of 37 clinical isolates of *T. asahii* were identified. Thirty-three (83.8%) isolates were from hospitalized individuals, with 81.82% collected in ICUs, an average hospital stay of 35 days, and a mortality rate of 51.6%. The tested strains displayed the largest mean inhibition zone for 2% sodium hypochlorite, indicating lower tolerance. A high level of efflux pump expression was detected among clinical isolates. Biofilm formation was detected in 25/67.5% of the isolates. Conclusions: These findings highlight the clinical relevance of *T. asahii*, particularly in critically ill individuals, and underscore the pathogen’s ability to tolerate biocides, express efflux pumps, and form biofilms, all of which may contribute to its persistence and pathogenicity in hospital environments. Enhanced surveillance and effective microbial control measures are essential to mitigate the risks associated with *T. asahii* infections.

## 1. Introduction

*Trichosporon* spp. belongs to the phylum Basidiomycota and comprises urease-positive, non-encapsulated microorganisms with yeast-like characteristics [1]. The genus has been revised several times in recent decades, driven by advances in the molecular phylogeny of basidiomycetous yeasts [2]. According to the classification proposed by Liu et al. (2015) [3], the order Trichosporonales comprises two families: Tetragoniomycetaceae and Trichosporonaceae. The latter includes the genera *Trichosporon*, *Apiotrichum*, *Cutaneotrichosporon*, *Effuseotrichosporon*, and *Haglerozyma*.

These fungi are widely distributed in the environment, being found in various habitats such as soil, water, and the microbiota of animals, including humans, where they colonize the skin and gastrointestinal tract [4]. They are etiological agents of serious healthcare-associated infections (HAIs), which are increasingly prevalent in intensive care unit (ICU) settings [5]. The prolonged hospitalization associated with ICU care significantly increases the likelihood of acquiring such infections, including those caused by *Trichosporon* species like *Trichosporon asahii* [6,7]. Among the most clinically relevant, *T. asahii* stands out as the main agent of invasive trichosporonosis, frequently isolated in neutropenic individuals and those undergoing immunosuppressive therapies [1,8]. Additionally, fungal colonization is common among these individuals, further increasing the risk of infection [9].

The pathogenicity of these species is directly associated with virulence factors, which facilitate the invasion and establishment of the fungus in the host’s tissues [10]. Biofilm is a polymeric extracellular matrix formation that facilitates fungal adherence to epithelial cells and evade host defense mechanisms, particularly phagocytosis [11]. Hemolysins, proteases, and lipases allow destabilization of host membranes and degradation of host connective tissues, cleavage of host immunity-associated proteins, ultimately aiding in the acquisition of nutrients and the escape from the host immune system [12,13].

As responsible for most cases of trichosporonosis, *T. asahii* represents a growing concern for public health. The clinical manifestations from this agent infections vary based on the affected tissue, and may include fever, brain and liver abscesses, endophthalmitis, fungemia, and respiratory or urinary tract infections [14,15,16]. This fungus is most isolated from blood, particularly in cases of fungemia and disseminated infections. It has also been recovered from other clinical specimens, including urine, especially in individuals with indwelling urinary catheters and skin lesions, reflecting its ability to colonize and invade multiple body sites [17,18], may act as an opportunistic pathogen, and can cause disseminated and invasive infections, especially in immunocompromised individuals, with reported mortality rates ranging from 30% to 90% [1,14,19].

The most common risk factors associated with *Trichosporon* infections include the use of broad-spectrum antibiotics, invasive medical procedures such as urinary catheterization and central venous catheterization, prolonged stays in intensive care units (ICUs), and underlying health conditions [16,17].

*Trichosporon* infections, particularly *Trichosporon* fungemia, exhibit a remarkably high 30-day mortality rate when compared to other fungal infections. Reported mortality rates range from 50% to 80%, varying according to patient population and underlying clinical conditions [20,21,22], which is significantly higher than many other fungal infections such as those caused by *Candida* spp. [23,24]. Possible reasons for this elevated mortality include the immunocompromised status of individuals, particularly those with hematological malignancies, the presence of central venous catheters, and the pathogen’s resistance to conventional antifungal treatments [20,21,22].

This scenario highlights the importance of the rational use of biocides in clinics and hospitals, emphasizing the use of products with proven efficacy against a broad spectrum of microorganisms, including species of the genus *Trichosporon*, especially *T. asahii.* The occurrence of infections in healthcare environments may be also related to the use of improper techniques for cleaning and disinfecting surfaces and inadequate waste management in healthcare services. Hand hygiene is a critical aspect of infection control and prevention in healthcare settings. By consistently implementing hand hygiene practices, using personal protective equipment (PPE) such as masks and gloves, and adhering to individual isolation protocols, healthcare facilities can significantly reduce the risk of HAIs [25,26].

**Figure 1:**
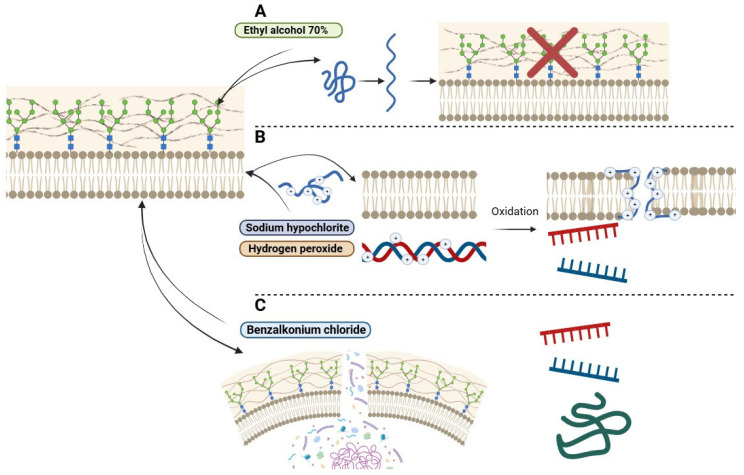
Mechanisms of action of biocides analyzed in this study: ethyl alcohol, sodium hypochlorite, hydrogen peroxide, and benzalkonium chloride. Legend: (**A**) probable action of ethyl alcohol in protein denaturation of the cellular wall; sodium hypochlorite and hydrogen peroxide; (**B**) and benzalkonium chloride (**C**) on Trichosporonales, demonstrating varying degrees of efficacy in disrupting biofilms, cell membranes, and intracellular components. red “X” means that the cell wall loses its functionality after the action of alcohol.

The main biocides used in healthcare settings include ethyl alcohols, hydrogen peroxide, quaternary ammonium compounds, and active chlorine release agents, each with different mechanisms of action and practical applications. Alcohol acts by denaturing microbial cell wall proteins, providing rapid and effective disinfection with ease of use. Hydrogen peroxide generates hydroxyl free radicals that damage membrane lipids, DNA, and other essential cellular structures. Quaternary ammonium compounds, although slightly corrosive and toxic, inactivate energy-producing enzymes, denature proteins and disrupt cell membranes; however, their effectiveness is reduced in the presence of organic matter. Active chlorine compounds, particularly sodium hypochlorite, are inexpensive and fast-acting disinfectants that oxidase cell proteins, making them widely used for surface cleaning in hospitals. However, their strong odor, volatility and corrosive nature, particularly at concentrations between 0.02% and 1.0%, pose challenges including material degradation and mucosal irritation [27] (Figure 1).

Biocide tolerance in fungi, particularly in the genus *Trichosporon*, can be attributed to several mechanisms, such as the expression of efflux pumps that actively transport undesirable compounds out of the cell, reducing the internal concentration of biocides and mitigating their lethal effects [28]. Additionally, biofilm formation acts as a protective barrier that hinders the tolerance diffusion of biocides and other compounds, such as antifungals [29]. Collectively, these mechanisms contribute to the adaptation and survival of *Trichosporon* spp. and other microorganisms on abiotic surfaces, particularly in healthcare settings (furniture, instruments, medical equipment, among others), and biotic surfaces (skin and mucous membranes of individuals and healthcare professionals) [30].

The aim of this study was to analyze individuals’ clinical characteristics, and evaluate tolerance to biocides commonly used in hospitals, as well as biofilm formation and efflux pump activity in isolates of *Trichosporon asahii*. This way, the study provides insights to improve infection prevention and control strategies in hospital and healthcare service environments.

## 2. Materials and Methods

This descriptive, experimental, and cross-sectional study analyzed 37 clinical non-duplicated isolates of *T. asahii* from both hospitalized and non-hospitalized individuals. The samples were collected by a clinical microbiology service in Juiz de Fora, Minas Gerais, Brazil, between January 2020 and December 2023.

The hospital involved in the study is a private healthcare facility with approximately 160 beds, including specialized units such as adult and neonatal intensive care units, a coronary care unit, a neurological unit, general wards, a surgical center, and outpatient services.

This study was conducted with the informed consent of all participants, in accordance with the project approved by the Ethics Committee for Research Involving Human Beings of the Federal University of Juiz de Fora, under CAAE 18611019.6.0000.5147 (approved on 19 December 2019).

### 2.1. Individuals

The inclusion criteria of this study consisted of individuals who had a positive culture result for *T. asahii*, regardless of age, sex, or origin (hospital-acquired or community-acquired). Multiple samples from the same individual were excluded.

### 2.2. Analysis of Medical Records

The medical records of individuals with *T. asahii* isolates were reviewed. Data collected included age, gender, length of hospitalization, patient origin (hospitalized or non-hospitalized), clinical specimen type and clinical outcome. This information was obtained directly in electronic medical records, and an electronic spreadsheet was used to record this data.

### 2.3. Assessment of the Integrity of Isolates

The samples from the collection were identified and preserved in sterile 2 mL vials with distilled water, also sterile, according to the method described by Diogo et al. (2005) [31]. Clinical isolates of *T. asahii* from the collection were cultured on Sabouraud Dextrose Chloramphenicol Agar (Neogen of Brazil, Lansing, MI, USA) and incubated at 35 °C for 48 h. Morphological characteristics were observed, and the Gram staining method was performed to assess the growth, viability, and purity of the isolates.

### 2.4. Identification of Yeasts

After incubation, the 37 isolates were identified by biochemical and physiological methods using the Vitek 2^®^ system (bioMérieux, Marcy-l’Étoile, France), according to the manufacturer’s instructions. The standard strain *T. asahii* ATCC 90039 (American Type Culture Collection, Manassas, VA, USA) was used as quality control, demonstrating satisfactory performance: 99.9% similarity.

### 2.5. Assessment of Tolerance to Biocides

For the test to assess tolerance to biocides commonly used in hospital environments, an adaptation was made to the disc diffusion technique described by *Clinical and Laboratory Standard Institute* (CLSI) [32]. Starting with suspensions of the isolates on a 0.5 McFarland turbidity scale, the isolates were evenly inoculated using a swab onto Mueller–Hinton agar (Kasvi, Pinhais, Brazil), where filter paper disks were added, soaked with 5 μL of 1%, 1.5%, and 2% sodium hypochlorite solution (Start, São Paulo, Brazil); 4.25% hydrogen peroxide diluted (Rioquímica, São José do Rio Preto, Brazil); 5% Benzalkonium chloride (Êxodo cientifica, São Paulo, Brazil); and ethyl alcohol 70% (Everest, Rio de Janeiro, Brazil). Duplicates were made for each concentration and the control. Inhibition zones around the discs were measured after 24 h of growth at 35 °C. The standard strain of *T. asahii* ATCC 90039 was used as the negative control.

### 2.6. Evaluation of Efflux Pump Activity

The expression of efflux pumps was assessed using ethidium bromide (EtBr) (Ludwig, Porto Alegre, Brazil), according to the method described by Cartwheel et al. (2011) [33] with modifications. The Mueller–Hinton agar (Kasvi, Pinhais, Brazil) was prepared by adding ethidium bromide at concentrations of 0.5 µg/mL, 1.0 µg/mL, 1.5 µg/mL, 2.0 µg/mL, and 2.5 µg/mL, and plates containing only the Mueller–Hinton agar (Kasvi, Pinhais, Brazil) medium were used as the control for microbial growth. Colonies of *T. asahii* from this culture were inoculated to obtain a suspension in sterile saline solution (Sanobiol, Pouso Alegre, Brazil) on the 0.5 MacFarland turbidity scale and inoculated using a Steers replicator into the culture medium. Duplicates were made for each concentration and control. The plates were incubated for 24 h at 35 °C, protected from light. After incubation, the plates were read under ultraviolet light, and the presence of fluorescence emitted by the samples was a negative indication of the action of the efflux pump. A standard strain of *T. asahii* ATCC 90039 was used as the negative control.

### 2.7. Detection of Biofilm Formation

Biofilm formation was detected using the adherence test method proposed by Pramodhini et al. (2021) [34]. Colonies of *T. asahii* previously grown on Sabouraud dextrose chloramphenicol agar (Neogen of Brazil, Lansing, MI, USA) were inoculated into 2 mL of a brain–heart infusion (BHI) broth (Oxoid, Basingtoke, UK) in test tubes and incubated at 35 °C for 48 h. After the incubation period, Safranin dye (Iodontosul, Porto Alegre, Brazil) was added to the test tubes and the adhesion of the yeast cell mass to the bottom and walls of the tubes was checked. The positive result was attributed to samples with clumps stained by safranin adhered to the surface of the tube. Standard strain of *T. asahii* ATCC 90039 was used as the negative control.

### 2.8. Statistical Analysis

Descriptive statistical analysis was performed, including percentage, absolute frequency, range and mean values for individuals’ age. A Students’ test-*t* and test-F were applied to the results of the mean inhibition halo diameters obtained around the disks impregnated with biocides. A *p*-value of 0.05 was considered, with the null hypothesis stating that there are no statistical differences between the mean inhibition halos of the groups. An odds ratio (OR) was calculated to assess the association between mortality and the expression of virulence factors.

## 3. Results

Among the 37 isolates non-duplicated analyzed, the majority were obtained from male individuals (29/78.4%). Most individuals were over 60 years old (22/59.5%), followed by those aged 13–59 years (12/32.4%) and ≤12 years (3/8.1%), with a mean hospitalization time of 35 days (range: 1–102 days). Clinical specimens were predominantly collected in hospital settings, especially in the neurological intensive care unit (11/29.7%) and inpatient units (9/24.3%).

Urine was the most frequently isolated specimen (21/56.7%), followed by bronchoalveolar lavage (10/27.1%). Of the 31 individuals with documented clinical outcomes, 51.6% resulted in death, highlighting the high mortality rate and clinical severity associated with *T. asahii* infection, particularly among critically ill hospitalized individuals (Table 1). Among the individuals who progressed to death, most isolates were obtained from urine (15/93.8%), with an invasive isolation pattern identified. In addition, the majority of these individual’s required intensive care support (13/16), and the mean age was 67 years, suggesting that advanced age and critical clinical condition may have contributed to the unfavorable outcomes.

**Figure 2:**
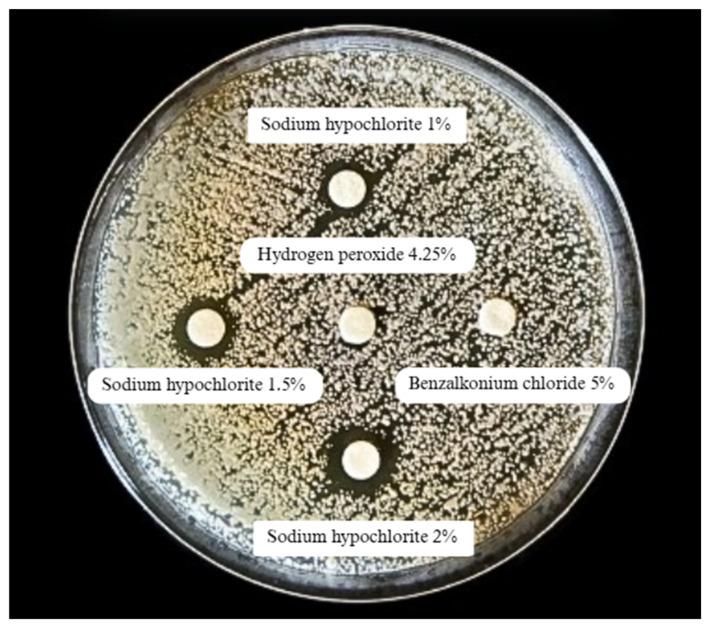
Disc diffusion test for determining biocide tolerance in *Trichosporon asahii.* Inhibition halos formed around discs containing varying concentrations of sodium hypochlorite. No inhibition halos were observed for benzalkonium chloride and hydrogen peroxide, indicating tolerance to these biocides at the tested concentrations.

Figure 2 represents the biocide tolerance determination test. As shown in Table 2, the clinical 37 isolates of *T. asahii* exhibited smaller inhibition halos than the reference strain *T. asahii* ATCC 90039 (n = 2) in response to most of the tested biocides, indicating greater tolerance. Statistically significant differences were observed with 70% ethanol (*p* = 0.00), 4.25% hydrogen peroxide (*p* = 0.00), and 5% benzalkonium chloride (*p* = 0.00), which exhibited significantly smaller mean halos among the clinical isolates. The F-test produced a value of 1.5 × 10^−17^ or 0.00, strongly indicating that the differences observed between the groups are statistically significant and not attributable to random variation. Although there was a marked difference in the mean diameter of the halos with 2% sodium hypochlorite (16.4 ± 6.01 mm with the clinical isolates versus 32.5 ± 10.61 mm with the ATCC strain), this difference was not statistically significant (*p* = 0.17), possibly due to the small sample size of the reference strain. These results suggest that clinical isolates of *T. asahii* are more tolerant of certain biocides, which could be significant for infection control in hospitals.

The biocides used in this study were classified according to their pH characteristics (Table 3). Sodium hypochlorite was alkaline, hydrogen peroxide was acidic, and both ethyl alcohol and benzalkonium chloride were neutral-to-slightly acidic or alkaline. This classification supports the interpretation of *T. asahii* responses under different chemical conditions [27].

In general, tolerance to biocides does not appear to be related to the acid–base behavior of these compounds or at least, there is no scientific evidence yet supporting such an association, especially in fungi. The efficacy of biocides depends on multiple factors, such as product quality, proper dilution, correct application, and spectrum of activity compatible with the institution’s epidemiological profile, as well as microbial characteristics [27].

The combined use of two or more agents should also consider the type of surface: biotic or abiotic, and, in this context, the specific material. Sodium hypochlorite, for example, is not recommended for metallic or stainless-steel surfaces due to the risk of corrosion. Likewise, it is not recommended for hand antisepsis and related uses [27,28].

**Figure 3:**
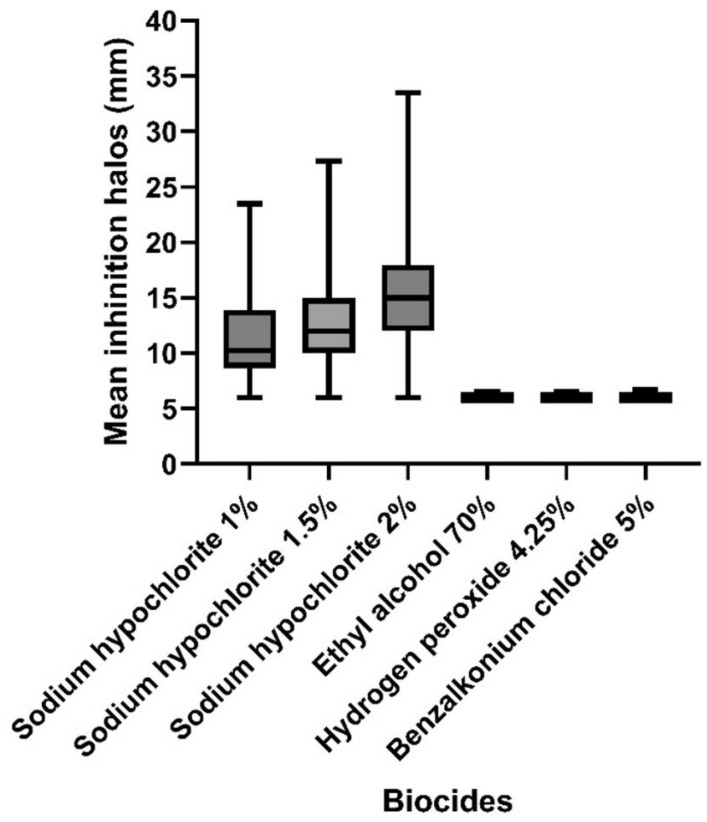
Distribution of the mean inhibition halos (in mm) produced by *Trichosporon asahii* isolates when exposed to various biocides. Legend: X—biocide compounds tested through disk diffusion; Y—mean inhibition halos in mm.

Biocides based on sodium hypochlorite (1%, 1.5%, and 2%) produced larger inhibition halos with greater variability, especially at the 2% concentration. This indicates higher efficacy against the tested isolates. In contrast, 70% ethyl alcohol, 4.25% hydrogen peroxide, and 5% benzalkonium chloride resulted in significantly smaller halos with low variability, suggesting lower antifungal activity or higher tolerance of the isolates to these agents. These results are consistent with those presented in Table 2, which reinforce that certain biocides, particularly chlorine-based compounds, demonstrate greater effectiveness in inhibiting the growth of *T. asahii* (Figure 3).

When comparing the inhibition halos formed by 37 isolates of *T. asahii* against 1% sodium hypochlorite and other biocides, the results revealed that the halos formed by 2% sodium hypochlorite were significantly larger (16.4 ± 6.01 mm) than those formed by 1% sodium hypochlorite (11.4 ± 4.34 mm), with a statistically significant difference (*p* = 0.000006609). Upon comparisons between 1% hypochlorite and the other biocides, ethyl alcohol 70%, 4.25% hydrogen peroxide, and 5% benzalkonium chloride, also revealed statistically significant differences (*p* < 0.00000001). The latter three biocides showed smaller inhibition halos (~6.1 mm), indicating the isolates’ greater tolerance or lesser efficacy. There was no statistically significant difference between 1% and 1.5% hypochlorite, which yielded an extremely low F-value (7.81 × 10^−55^, or 0.22), suggesting that the difference between these two concentrations is not statistically significant. These data reinforce the idea that sodium hypochlorite, especially at higher concentrations, has a greater inhibitory capacity against *T. asahii* and is the most effective biocide among the compounds evaluated, in other words, indicating lower tolerance (Table 4).

The efflux pump activity assay demonstrated a positive expression rate in all clinical isolates of *T. asahii* at EtBr concentrations of 0.5, 1.5, 2.0, and 2.5 µg/mL. At 1.0 µg/mL, three isolates (8.1%) did not exhibit efflux pump activity, while the remaining 91.9% remained positive. These results indicate a consistently high level of efflux pump expression among clinical isolates, potentially contributing to their tolerance against antimicrobial agents (Table 5).

Observing the relationship between the production of virulence factors and possible influence on mortality outcome, and considering *T. asahii* isolates, the biofilm formation had an odds ratio (OR) of 0.47 with a 95% confidence interval (CI) of 0.11 to 2.11, while efflux pump expression had an odds ratio of 2.13 with a 95% CI of 0.17 to 26.04. In other words, an OR < 1 suggests that biofilm formation may not be associated with the risk of death among the individuals evaluated, whereas an OR > 1 indicates a possible positive correlation between efflux pump expression and mortality (Table 6).

Table 7 shows a comparative analysis between the virulence factors—biofilm formation, expression of efflux pumps, and tolerance to biocides—and the clinical outcomes of individuals classified as non-hospitalized individuals, hospital-discharged individuals, and individuals with hospital death. Biofilm formation was more prevalent in the hospital discharge group (29.7%), followed by individuals who died (27.1%).

Efflux pump expression was positive in 40.5% of individuals who died and 35.2% of those who were discharged from the hospital. Tolerance to three biocides was higher in the group of individuals with hospital death (37.8%), followed by the hospital discharge group (32.4%) and, lastly, the non-hospitalized group (10.8%), especially for three biocides. The combination of all the virulence factors (biofilm + efflux pump + tolerance to biocides) was more frequent among the individuals who died (24.3%), showing tolerance to three biocide concentrations tested (Table 7).

In addition to the frequency of *T. asahii* and hospitalization, we observed clinical characteristics associated with isolates that strongly expressed the efflux pump and had a high capacity for biofilm formation, though these associations were not statistically significant. Most individuals were male (90.5%) and over 60 years old (71.4%), with an average age of 62, suggesting a possible relationship between biofilm formation and age extremes. This relationship may be due to a senescent or immature immune response. The main clinical specimens were urine (52.4%) and bronchoalveolar lavage fluid (33.3%). The clinical outcome was unfavorable in 42.9% of cases.

The microbiological and clinical characteristics varied according to the individuals’ clinical outcomes. Among the 16 individuals who died, the mean age was 67 years, with a predominance of males (14/87.5%). Urine was the main source of clinical isolates (15/93.7%), followed by bronchoalveolar lavage (1/6.3%), and biofilm formation was detected in 10 (62.5%) of the strains.

For the 15 individuals who were discharged from hospital, the mean age was 49 years, and 73.3% were male. Bronchoalveolar lavage was the most frequent sample type (9/60.0%), followed by urine (4/26.6%) and tracheal aspirate (2/13.3%). Biofilm production was observed in 11 (73.3%) of the isolates.

Among the six non-hospitalized individuals, the mean age was 54 years, with males accounting for 66.6% of cases. The isolates were mainly obtained from skin and nail scrapings (3/50.0%), urine (2/33.3%), and scalp hair (1/16.3%). Biofilm formation was identified in three (50.0%) of the strains.

## 4. Discussion

In this study, the epidemiological and microbiological data presented show the clinical profiles of individuals with *T. asahii* infections (n = 37). There was a predominance of male individuals (78.4%) and individuals over 60 years of age (59.5%), which is consistent with other studies suggesting greater susceptibility among men, possibly due to immunological factors or more prevalent comorbidities in this group [35,36].

A study conducted at a hospital in Saudi Arabia [35], which evaluated the clinical epidemiology of *Trichosporon* spp. infections in microbiological cultures over a five-year period, showed that *T. asahii* was the predominantly isolated species, accounting for 90.5% of the cases, especially in male individuals, supporting the findings of the present study.

A retrospective cohort study between 2019 and 2023 that included all individuals with urinary isolates of *Trichosporon* spp. conducted at a tertiary medical center in Mexico City [36] identified 26 individuals with positive urine cultures for *T. asahii*. Of these, 19/73% were male, all individuals had urinary tract catheters, 25/96% had central venous catheters, and 18/70% had invasive mechanical ventilation and stayed in the intensive care unit (ICU).

Factors that may explain the male frequency of invasive fungal infections include differences in steroid hormone homeostasis, sex-specific immune responses, behavioral factors such as occupational exposures, medical comorbidities like human immunodeficiency virus (HIV), and gender disparities in health care [36,37]. A recent study successfully identified several *Trichosporon* species isolated from superficial infections in male individuals in central Brazil, highlighting the frequency of *T. asahii* (72%) among the isolates. In addition, the study emphasizes the clinical significance of *Trichosporon* species as having an opportunistic capability of causing superficial infections and potentially serious invasive disease, raising awareness of their medical importance [2].

The most frequently used clinical specimens for isolating the fungus were bronchoalveolar lavage (27%) and urine (56.7%), suggesting a predilection for respiratory and urinary tract infections.

An epidemiological study analyzed 140 cases of *T. asahii* infections reported over a 23-year period. These cases were documented across multiple continents, including Asia, Europe, North and South Americas, and Africa, with the highest incidence of *T. asahii* observed in Asia (77.1%). According to the article cited, the higher incidence of *T. asahii* infection in Asia is attributed to increased antibiotic use, invasive medical equipment, and chemotherapy among immunocompromised individuals, particularly those with blood diseases, which are more prevalent in this region compared to others. The study highlights significant regional variations in infection types and clinical outcomes. Among the 140 individuals with *T. asahii* infections, blood infection was the most common (47/140, 33.6%), followed by the urinary system (40/140, 28.6%), respiratory system (29/140, 20.7%), and integumentary system (21/140, 15.0%) [17].

HAI from *Trichosporon* spp. represents a significant challenge for healthcare systems, leading to elevated healthcare costs due to prolonged hospital stays, higher treatment expenses, and greater morbidity [1,38]. The results of this study highlight the severity of these infections in hospitalized individuals, with more than half (51.6%) of individuals experiencing an adverse clinical outcome: death. These findings emphasize the urgent need for infection prevention strategies and the improvement of therapeutic protocols, especially in hospital settings, where individuals’ health is compromised [6,8].

Of the 37 individuals analyzed with *T. asahii* infections, 31 (83.8%) were hospitalized, among whom 16 (51.6%) died in the hospital. Although the mortality rate is worrying, there is still a possibility of recovery, especially with early diagnosis and appropriate treatment. Previous studies suggest an unfavorable prognosis for these infections, particularly in individuals with severe immunosuppression [14,20]. Furthermore, a study of rare yeasts in Latin America reported a crude mortality rate of 40.8%, with an increased risk of death associated with fungemia and *T. asahii* infections [39].

Our findings did not allow us to establish a direct correlation between clinical outcome and the site of fungal isolation. All identified deaths (n = 16) occurred in individuals with *T. asahii* detected in urine (n = 15), followed by bronchoalveolar lavage. However, the medical records did not allow a clear distinction between colonization and invasive infection, making it impossible to identify variations in the prognosis of the patients evaluated.

Individuals who are hospitalized and remain in the ICU for extended periods face a heightened risk for *Trichosporon* spp. infections, particularly due to their compromised immune systems and the use of invasive medical devices. Overall, the association between prolonged ICU stays and *Trichosporon* spp. infections is a critical concern for patient management and outcomes.

The length of hospital stay (LOS) directly impacts hospitalization costs and the risk of acquiring healthcare-associated infections (HAIs), particularly those caused by multidrug-resistant organisms (MDROs). Extended LOS increases healthcare expenses due to the need for intensive care and complementary treatments. Studies indicate that antimicrobial-resistant infections can prolong hospital stays by an average of 18.8 days, adding approximately EUR 11,549 per individual [40].

In this study, the disk diffusion technique was used to evaluate the tolerance profile to the biocides used in routine hospital decontamination. Although it is not a widely standardized method by reputable institutions, it is simple to perform, easily reproducible in clinical laboratories, and cost-effective. Despite being explored little in scientific literature, particularly in *Trichosporon* spp., it has great applicability.

The results showed significant variations in tolerance to the tested biocides, especially sodium hypochlorite, where the isolates showed the least tolerance, with considerably larger inhibition halos at concentrations of 1.5% and 2%. The standard strain of *T. asahii* ATCC 90039 exhibited average inhibition halos of 32.5 mm, while the clinical isolates had an average of 16.4 mm. The larger inhibition halo observed for the standard strain compared to the halos of the isolates (expected result) can be attributed to the absence of continuous exposure to biocides in the standard strain.

The isolates exposed to the compound hydrogen peroxide diluted to 4.25%, benzalkonium chloride at 5%, and ethyl alcohol at 70% exhibited reduced halos (average of 6.1 mm) for both tested groups, which suggests greater tolerance of *T. asahii* to these biocides. This tolerance is particularly concerning, as these compounds are widely used as disinfectants/antiseptics in both clinical and hospital environments, as well as in domestic settings. Therefore, based on our results, we can infer low tolerance for the compounds benzalkonium chloride at 5%, hydrogen peroxide diluted to 4.25%, and ethyl alcohol 70%, and greater efficacy of sodium hypochlorite at 2% among the clinical isolates of *T. asahii* in this study.

It is imperative to ensure the accurate dilution of biocides and manage surface cleaning techniques effectively. This highlights the necessity for ongoing training of cleaning personnel in hospitals and other healthcare environments. Regular monitoring and surveillance are critical for maintaining the efficacy of biocides in the prevention of hospital-acquired infections. The effectiveness of disinfectants is significantly contingent upon their proper application; incorrect usage can lead to insufficient decontamination, thereby increasing the risk of nosocomial infections [30].

According to Brazilian and European guidelines [27,41], several compounds are classified as broad-spectrum biocides, namely quaternary ammonium compounds, hydrogen peroxide, and sodium hypochlorite. The recommendations for their application in clinical and hospital environments define specific concentrations and instructions for use, playing a vital role in infection control and maintaining health safety. Ethyl alcohol 70% acts by denaturing the cell wall proteins of microorganisms and is effective in disinfecting surfaces, sanitizing hands, and preventing superficial fungal infections. Sodium hypochlorite acts by oxidizing cell proteins and is widely used to clean hospital surfaces, although it can damage certain materials at concentrations between 0.02% and 1.0%. Hydrogen peroxide destroys microbial cells by forming free radicals that damage lipids, proteins, and DNA, and is used to disinfect surfaces and treat wounds. Benzalkonium chloride ruptures the cell membrane of microorganisms, releasing cytoplasmic components and degrading proteins and nucleic acids, resulting in cell death [27,41].

Studies on the tolerance of *T. asahii* to biocides are scarce, resulting in a significant gap in the understanding of their adaptive capacity to these chemical agents. Despite the importance of the genus in clinical and environmental contexts, there is a lack of consistent data on the efficacy of biocides against these yeasts and the possible mechanisms involved in tolerance, such as alterations in the cell membrane or the presence of efflux systems.

In the present study, the tested strains exhibited the largest mean inhibition zone for 2% sodium hypochlorite, indicating lower tolerance. However, the low tolerance of widely used compounds such as 4.25% diluted hydrogen peroxide, 5% benzalkonium chloride, and ethyl alcohol 70% highlights the need to review hospital disinfection practices to ensure adequate control of infections by this pathogen. More effective disinfection strategies, based on biocides with greater antifungal activity, should be implemented in hospital environments to minimize the risk of tolerance to these biocides.

Sodium hypochlorite is widely used as a disinfectant in clinical environments, but its use is limited by several factors. Its high reactivity can lead to corrosion of medical equipment and delicate surfaces. In addition, its volatility and release of chlorine gas can cause respiratory and eye irritation, posing health risks to staff and individuals [41].

Studies involving other emerging fungi, such as *Candida auris*, have shown concern about microbial tolerance to disinfectants. Clinical strains of *C. auris* have demonstrated tolerance to chlorine (0.01–0.02 mg/L) and benzalkonium chloride (16–32 mg/L). Additionally, *C. auris* has the ability to form biofilms, which further reduces the effectiveness of biocides. This highlights the need for the careful selection and use of biocides in healthcare settings to effectively control microorganisms [42].

Another study assessed biocide tolerance using the disk diffusion technique, finding that clinical isolates of *Candida* spp. exhibited greater tolerance to biocides compared to the reference strains. Sodium hypochlorite was identified as the most effective biocide, particularly at a concentration of 2%, which is crucial for infection control practices in healthcare settings, while hydrogen peroxide and benzalkonium chloride showed no measurable inhibition zones, indicating tolerance across all tested groups [43].

In addition to fungi, studies with multidrug-resistant hospital bacteria also corroborate the need for a more careful approach to the choice of biocides. A study by Dias et al. (2017) [44] assessed the tolerance profiles of clinical isolates of *Pseudomonas aeruginosa* and *Acinetobacter baumannii* to biocides used in hospital. The researchers found that carbapenem-resistant isolates exhibited greater tolerance to sodium hypochlorite 2% and benzalkonium chloride 5% compared to carbapenem-susceptible strains. Regarding infection control management in hospitals, these findings suggest that the use of benzalkonium chloride may be ineffective in controlling these microorganisms, due to their tolerance to the compound. This shows that the same product may be effective against certain microorganisms but not others that also colonize the hospital environment.

The compounds evaluated in this study are routinely used in this hospital. The greater tolerance shown by these *T. asahii* isolates to biocides such as hydrogen peroxide and benzalkonium chloride may be associated with the selective pressure resulting from exposure to these disinfectants in the hospital environment.

Thus, these studies suggest that standardized disinfection strategies may be insufficient given the complexity of today’s hospital microbiota. And they point to a common challenge: the adoption of more specific protocols that take into account not only the presence of multidrug-resistant microorganisms, but also the investigation of the molecular mechanisms involved in biocide tolerance, which can compromise biosafety in clinical environments.

The evaluation of the phenotypic efflux pump mechanism revealed a high positivity rate for all isolates at four tested concentrations: 0.5 µg/mL, 1.5 µg/mL, 2.0 µg/mL, and 2.5 µg/mL. Only three isolates of *T*. *asahii* did not express this mechanism at a concentration of 1.0 µg/mL. Efflux pumps, a common mechanism underlying antimicrobial resistance, allow fungi to expel toxic substances, including antifungal drugs, thereby preventing their therapeutic action [45]. The high frequency of efflux pump activity in *T. asahii* isolates suggests adaptation to environments with selective pressures, such as those involving prolonged antifungal use [46]. The expression of this process is regulated by a few genes, including *CDR2*, *CDR1,* and *MDR1*, which regulate the synthesis of ABC transporter proteins. These proteins operate at the cell membrane, actively exporting azole compounds out of the cell. When these genes are overexpressed, the production of ABC transporters increases significantly, leading to enhanced efflux pump activity. This heightened activity leads to a greater export of azole substances from the cell, thereby contributing to resistance [47].

In this study, the ethidium bromide technique was used, which is still little used in the analysis of fungi. A recent study investigated the mechanisms of resistance and assessed the activity of efflux pumps in clinical isolates of *Candida*. The results indicated that the overexpression of genes related to efflux pumps (*MDR1*, *CDR1,* and *CDR2*) represents a relevant factor in the resistance observed in clinical isolates of *Candida* spp. This multifactorial mechanism of resistance hinders the effectiveness of available treatments and reinforces the need to develop therapeutic strategies that specifically target these efflux pathways [43].

Efflux pumps can expel both biocides and drugs simultaneously, potentially compromising therapeutic success and complicating efforts to contain the spread of resistant microorganisms. This finding is particularly concerning, as it reinforces the hypothesis that infections caused by *T*. *asahii* may be challenging to treat due to resistance mediated by these pumps. In this context, the use of efflux pump inhibitors represents a promising strategy to enhance the efficacy of antifungal drugs in future treatments [45,46].

The association between efflux pump expression and the risk of death, as indicated by an OR of 2.13, suggests that efflux pump may contribute to virulence and potentially the severity of *T. asahii* infections. In contrast, biofilm formation was associated with a lower OR (0.47), which may imply a negative correlation with mortality in this study.

Biofilm formation was detected in 25/67.6% of *T. asahii* isolates. Biofilm formation, especially on medical devices, is considered a critical strategy for yeast persistence in hospital environments. Biofilm confers increased resistance to the host immune system and antifungal treatments, making it difficult to establish appropriate antifungal therapy and manage the patient, especially in hospital settings [39,48]. Studies evaluating biofilm expression in clinical isolates of bacteria and fungi are still incipient. However, the available literature points to a similar role, greatly contributing to antimicrobial resistance, biocide tolerance, and immune system evasion. A multicenter study reported that 82.9% of individuals with *Trichosporon* fungemia had central venous catheters, emphasizing the healthcare-associated nature of these infections [20]. Hematological diseases (47.7%) and postoperative status (34%) were the main clinical conditions observed. Among postoperative individuals, thoracic (43.3%) and abdominal (40%) surgeries were the most frequently reported, while in solid organ transplant recipients, kidney (57.1%), heart (28.5%), and lung (14.3%) transplants were associated with the onset of fungemia. The 30-day mortality rate was remarkably high, reaching 51.1% [20]. We underscore the role of biofilm formation by these organisms to colonize medical devices and corroborate for bad prognosis in HAI individuals.

The combination of the three mechanisms—biofilm formation, expression of efflux pumps, and tolerance to biocides—was present in nine of the cases that died and was associated with tolerance to all biocides tested, suggesting a synergistic effect that could contribute to therapeutic failure and unfavorable outcomes. These findings reinforce the importance of phenotypic characterization of clinical isolates in terms of resistance and virulence mechanisms, as a strategic tool for appropriate clinical management and prevention of serious complications, especially in hospital settings.

The factors that facilitate adhesion and biofilm formation by *Trichosporon* spp. have not yet been determined [45,49]. *Trichosporon* spp. can grow in yeast and arthroconidia forms. It has been proven that arthroconidia is important in biofilm formation by *T. asahii*, but biofilms formed by yeast cells have also been found [48]. Thus, biofilm formation represents a significant virulence factor since it increases microbial tolerance to disinfectants/antiseptics and contributes to the persistence of these microorganisms on various surfaces [28].

This study presents some limitations that should be considered when interpreting the results. The identification of *T. asahii* strains was performed using a phenotypic method (Vitek 2^®^ system), which, although widely used in clinical practice, does not have the same accuracy as molecular biology-based techniques for species differentiation within the genus. Furthermore, the disk diffusion method used to assess biocide tolerance, while simple and cost-effective, is not yet widely standardized by international organizations for use with yeasts, which may limit the comparability of the results with other studies. The absence of the molecular evaluation of resistance mechanisms, such as gene expression related to efflux pumps or biofilm formation, also limits a deeper understanding of the resistance observed.

## 5. Conclusions

*T. asahii* infections present a serious challenge to healthcare systems, associated with high mortality rates and considerable resistance mechanisms. Our findings demonstrate that 2% sodium hypochlorite is more effective against *T. asahii* isolates when compared to compounds widely used in disinfection/antisepsis routines, such as 70% ethyl alcohol, 4.25% hydrogen peroxide, and 5% benzalkonium chloride. Furthermore, the finding of 67.5% total clinical isolates forming biofilms and exhibiting high level of efflux pump expression among clinical isolates highlights the adaptive capacity of these yeasts, complicating clinical treatment and outbreak containment efforts. The results obtained in this study reinforce the need for hospitals to prioritize the use of more effective biocides, especially those with a broad spectrum of action against healthcare-associated pathogens, supported by robust scientific evidence. Furthermore, the implementation of continuous surveillance of biofilm formation and biocide tolerance in clinical isolates is fundamental to guide effective infection control strategies. The importance of regular training for healthcare professionals on the proper use and limitations of antiseptic and disinfectant agents is also emphasized. Thus, the adoption of stringent microbial control measures is essential to reduce the risks associated with *T. asahii* infections.

## Figures and Tables

**Table 1:** Epidemiological and microbiological aspects of hospitalized and community-dwelling individuals with a *Trichosporon asahii* infection (n = 37).

Clinical and Epidemiological Parameters		*Trichosporon asahii* (n = 37)
Gender: n (%)		
	Female	8 (22.6)
	Male	29 (78.4)
Age: n (%)		
	≤12 years	3 (8.1)
	13–59 years	12 (32.4)
	≥60 years	22 (59.5)
Length (in days) of hospitalization: average (range)		35 (1–102)
Collection unit: n (%)		
	Surgical center	5 (13.5)
	Non-hospitalized	6 (16.2)
	Coronary unit	1 (2.7)
	Inpatient unit	9 (24.3)
	General ICU	3 (8.1)
	Neurological ICU	11 (29.7)
	Neonatal ICU	2 (5.4)
Clinical specimen: n (%)		
	Bronchoalveolar lavage	10 (27.1)
	Nail scraping	1 (2.7)
	Scalp hair	1 (2.7)
	Skin shave	2 (5.4)
	Tracheal aspirate	2 (5.4)
	Urine	21 (56.7)
Clinical outcome: n = 31		
	Hospital discharge	15 (48.4)
	Death	16 (51.6)

**Table 2:** Evaluation of tolerance to biocides among *Trichosporon asahii* isolates, means, and standard deviation, with the results in the halo patterns.

Biocide	Average Inhibition Halo Diameter (mm)		Test-*t* *S*tudent	Test-F *p* (F ≤ f)
	*Trichosporon asahii* (n = 37)	*T. asahii* ATCC 90039 (n = 2)		
Sodium hypochlorite 1%	11.4 (±4.34)	16.0 (±5.66)	0.45	0.40
Sodium hypochlorite 1.5%	13.1 (±5.31)	18.0 (±4.24)	0.33	0.86
Sodium hypochlorite 2%	16.4 (±6.01)	32.5 (±10.61)	0.27	0.17
Ethyl alcohol 70%	6.1 (±0.09)	9.0 (±1.42)	0.21	0.00
Hydrogen peroxide 4.25%	6.1 (±0.09)	8.5 (±0.71)	0.13	0.00
Benzalkonium chloride 5%	6.1 (±0.13)	7.5 (±0.72)	0.21	0.00

Legend: *p*-value of 0.05.

**Table 3:** Classification of biocides based on pH characteristics.

Biocide	pH Range	Classification
Sodium hypochlorite	pH 11–13	Alkaline
Ethyl alcohol	pH neutral to slightly acidic	Neutral/Slightly acidic
Hydrogen peroxide	pH 3.5–4.5	Acidic
Benzalkonium chloride	pH 6–8	Neutral/Slightly alkaline

**Table 4:** Comparison of halos between compounds of *Trichosporon asahii* isolates, means, and standard deviation.

Isolate	Biocide		Test-*t S*tudent	Test-F *p* (F ≤ f)
*Trichosporon asahii* (n = 37)	Sodium hypochlorite 1% 11.4 (±4.34)	Sodium hypochlorite 1.5% 13.1 (±5.31)		
			0.140954942	0.22
	Sodium hypochlorite 1% 11.4 (±4.34)	Sodium hypochlorite 2% 16.4 (±6.01)		
			0.000060699 *	0.04
	Sodium hypochlorite 1% 11.4 (±4.34)	Ethyl alcohol 70% 6.1 (±0.09)		
			0.00000000002 *	0.00
	Sodium hypochlorite 1% 11.4 (±4.34)	Hydrogen peroxide 4.25% 6.1 (±0.09)		
			0.000000002 *	0.00
	Sodium hypochlorite 1% 11.4 (±4.34)	Benzalkonium chloride 5% 6.1 (±0.13)		
			0.0000000000 *	0.00

Legend: *p*-value of 0.05. (*) There was a significant difference.

**Table 5:** Evaluation of positivity levels for efflux pump expression under different concentrations of ethidium bromide (Etbr) in clinical isolates of *Trichosporon asahii*.

Isolate	Etbr Concentration				
	0.5% (n/%)	1.0% (n/%)	1.5% (n/%)	2.0% (n/%)	2.5% (n/%)
*T. asahii* (n = 37)	37.0/100.0	34.0/91.9	37.0/100.0	37.0/100.0	37.0/100.0
*T. asahii* ATCC 90039 (n = 2)	2.0/100.0	2.0/100.0	2.0/100.0	2.0/100.0	2.0/100.0

**Table 6:** Odds ratio for the association between the outcome of death and the expression of virulence factors (biofilm formation and efflux pump expression) in *Trichosporon asahii* isolates.

OR (95% Confidence Interval)	
Biofilm formation	Efflux pump expression
0.47	2.13
(0.11–2.11)	(0.17–26.04)

Odds ratio with a 95% confidence interval: OR = 1, no correlation; OR > 1, positive correlation; OR < 1, negative correlation.

**Table 7:** Correlation between biofilm formation, efflux pump expression and biocide tolerance with the clinical evolution of individuals.

Virulence Factor	Non-Hospitalized Individuals (n = 06/16.2%)	Discharged Individuals (n = 15/40.5%)	Individuals with Hospital Death (n = 16/43.3%)
Biofilm formation	n = 3/8.1	n = 11/29.7	n = 10/27.1
Efflux pump expression	n = 6/16.2	n = 13/35.2	n = 15/40.5
Tolerance to biocides	n = 4/10.8 (3 biocides)	n = 12/32.4 (3 biocides)	n = 14/37.8 (3 biocides)
	n = 1/2.7 (*4 biocides)	n = 3/8.1 (*4 biocides)	n = 2/5.4 (*4 biocides)
	*Two concentration*	*One concentration*	*All concentrations*
Biofilm formation + efflux pump expression + Tolerance to biocides	n = 2/5.4 (3 biocides)	n = 6/16.2 (3 biocides)	n = 9/24.3 (3 biocides)
	n = 1/2.7 (*4 biocides)	n = 3/8.1 (*4 biocides)	n = 1/2.7 (*4 biocides)
	*Two concentrations*	*One concentration*	*All concentrations*

Legend: biocides tested—70% ethyl alcohol, 4.25% hydrogen peroxide, 5% benzalkonium chloride, and sodium hypochlorite (in three different concentrations: 1%, 1.5%, and 2%). (*) There was a significant difference.

## Data Availability

The authors declare that the data related to the medical records of the individuals who participated in this research are not available due to privacy restrictions or ethical considerations. The experimental data have been disclosed in this manuscript while respecting ethical constraints regarding the identification of these individuals.
